# Eumycetoma Osteomyelitis of the Calcaneus in a Child: A Radiologic-Pathologic Correlation following Total Calcanectomy

**DOI:** 10.1155/2015/129020

**Published:** 2015-09-21

**Authors:** Tamer Ahmed EL-Sobky, John Fathy Haleem, Shady Samir

**Affiliations:** Orthopedic Surgery Department, Faculty of Medicine, Ain Shams University, 38 Abbasia Square, Masjed Alnoor, Cairo, Egypt

## Abstract

Fungi are unusual causes of pedal osteomyelitis in children and adolescents. Eumycetoma is a chronic cutaneous and subcutaneous infection caused by various genera of fungi. A provisional diagnosis of foot mycetoma is made after clinical assessment. Radiologic-pathologic correlation is an essential supplement for the accurate diagnosis of osteoarticular infections. This paper aims to sensitize orthopedic surgeons, radiologists, and pathologists to the importance of correlative imaging findings in relation to surgical and microscopic pathology in osteoarticular infections, specifically eumycetoma osteomyelitis of the foot. From our review of the published data, the present case is the first report of radiologic-pathologic correlation in eumycetoma osteomyelitis of the calcaneus. This paper describes a case of eumycetoma osteomyelitis of the calcaneus in a child in which diagnostic X-rays and magnetic resonance imaging (MRI) were correlated with the surgical and microscopic pathologic features, for establishing an appropriate diagnosis and treatment. We conclude that there is a significant agreement between radiologic and pathologic evaluation for assessment of eumycetoma osteomyelitis of the calcaneus. Radiologic-pathologic correlation amplified our interpretation of imaging information available on plain radiographs and MRI and augmented diagnostic confidence. Similarly, anatomic-histopathological correlations consolidated diagnostic accuracy.

## 1. Introduction

Eumycetoma is an unusual chronic cutaneous and subcutaneous infection caused by various genera of fungi. Approximately 40% of mycetomas worldwide are eumycotic rather than actinomycotic (i.e., caused by bacterial actinomycetes). The disease is characterized by marked progressive destruction of soft tissue and bones causing functional disability. The most common site of occurrence is the foot [1]. A provisional diagnosis of foot mycetoma is made after clinical assessment. Some causal agents of mycetoma are difficult to identify by culture methods alone. Besides, clinical and histopathological examinations alone do not detect the spread of disease along the different tissue planes and bone and are not specific enough to identify the causative agent to the species level. Imaging techniques such as X-rays and MRI can aid in the early diagnosis of the lesion and can be used to determine the extent of lesions [2–4].

We assume that radiologic-pathologic correlation is an essential supplement for the accurate diagnosis and management of osteoarticular infections. From our review of the published data, the present case is the first report of radiologic-pathologic correlation in eumycetoma osteomyelitis of the calcaneus. The purpose of this review is to demonstrate why performing this correlation is an essential part of the diagnostic process of osteoarticular infections. This paper aims to sensitize orthopedic surgeons, radiologists, and pathologists to the importance of correlative imaging findings in relation to surgical and microscopic pathology in eumycetoma osteomyelitis of the foot. Our patient was informed that data concerning the case would be submitted for publication.

## 2. Case Report 

A 14-and-a-half-year-old boy from Fayoum Governorate in rural Egypt complained of an initially painless right foot swelling for the past six years. Multiple sinus tracts developed with an initial purulent discharge and an eventual extensive black granular discharge. He was previously subjected to multiple bony debridement procedures in other institutions to which the he did not respond. Based on the clinical/radiologic suspicion and results of culture and sensitivity from discharging sinuses that revealed Gram positive* Staphylococcus aureus*, the patient was diagnosed with nonspecific bacterial calcaneal osteomyelitis. The patient was treated with multiple courses of oral and intravenous antibiotics over the following six years, to which he did not respond. The patient walked with painful limp that deteriorated over the past years till he finally used crutches permanently and became a household ambulatory. The patient was nonimmunosuppressed and had no systemic manifestations or other skeletal complaints. Blood and serum chemistry were also unremarkable.

By examination, there was diffuse right foot oedema and tenderness especially over the hind foot. Two discharging sinuses were observed in relation to the hind foot. There was painful passive motion of the subtalar and ankle joints.

### 2.1. Radiologic-Pathologic Examination

**Figure 1:**
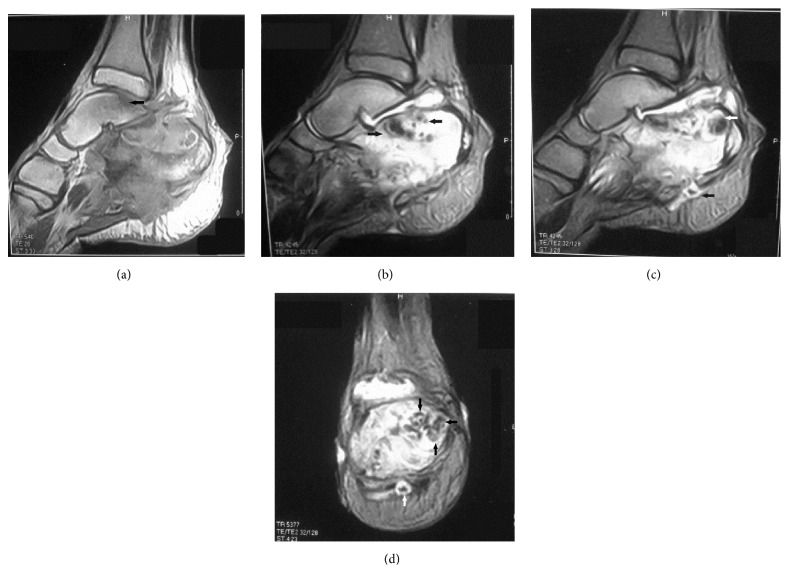
(a) T1-weighted sagittal MRI of the right foot. Note the altered marrow signals seen almost totally replacing the marrow texture of the calcaneus and to a lesser extent the opposing posteromedial aspect of the talus (black arrow) eliciting an abnormal low signal. (b) T2-weighted sagittal MRI of the right foot. Note the abnormal high signals replacing the marrow texture of the calcaneus. Note the multiple low-intensity lesions (black arrows) that may represent a conglomerate of grains in the background of diffuse high-intensity inflammatory bone changes. These low-intensity lesions are known as dot in circle. (c) T2-weighted sagittal MRI of the right foot. Note the low-intensity cavitary lesion of the posterior calcaneus (white arrow) that was found to correlate well to a conglomerate of black grains noticed in the gross pathologic specimen of the calcaneus. Note the same low-intensity lesion (black arrow) in the background of diffuse hyperintense inflammatory soft tissue changes (dot in circle). (d) T2-weighted coronal MRI of the right foot. Note the multiple small low-intensity lesions (black arrows) that may represent a conglomerate of grains in the background of diffuse hyperintense inflammatory bone changes. The same lesion is depicted in the soft tissue (white arrow).

X-ray findings of the foot and ankle revealed massive bony destruction of the calcaneus consistent with chronic osteomyelitis. MRI of the right ankle and foot was performed to characterize and evaluate the extent of the disease. Technique of examination included sagittal T1-, STIR, and T2-weighted images, axial T1- and T2-weighted images, and coronal T2-weighted images. The salient abnormality was the markedly altered marrow signals seen almost totally replacing the marrow texture of the calcaneus and to a lesser extent involving the opposing posteromedial aspect of the talus eliciting abnormal low T1, high T2, and iso-to-high STIR signals with evident cortical breaching and associated large soft tissue component eliciting low T1 and high T2 and STIR signals (Figures 1(a), 1(b), 1(c), and 1(d)).

**Figure 2:**
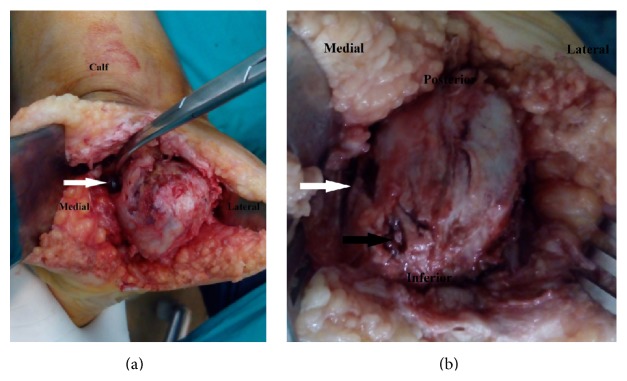
(a) Intraoperative images of calcaneus employing the heel splitting approach. Note the conglomerate of black grains arising from the cavity on the medial surface of calcaneus (white arrow). (b) Intraoperative images of calcaneus employing the heel splitting approach. Note the large medial cavity (white arrow) and cortical erosion covered by minute black grains (black arrow).

**Figure 3:**
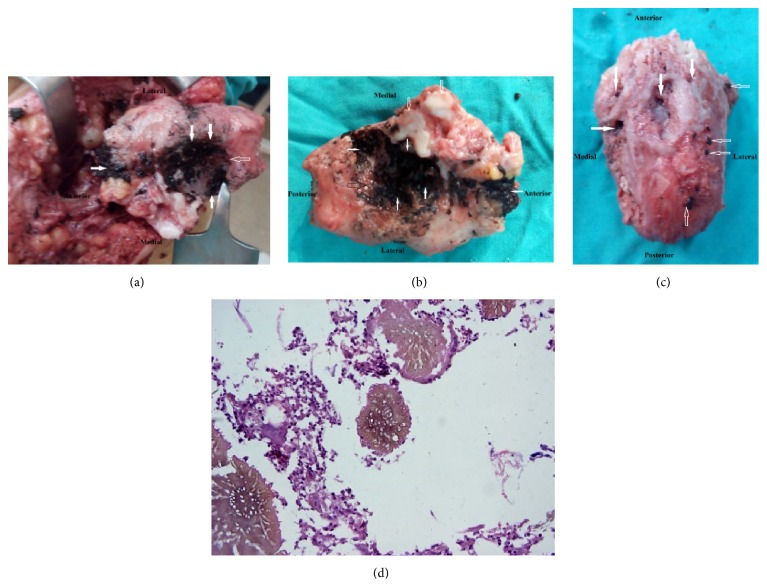
(a) Intraoperative images of superior surface of calcaneus employing the heel splitting approach. Note the extensive conglomerates of black grains scattered over that surface (solid arrows). Note the complete destruction of the articular cartilage of the posterior facet of the subtalar joint (hollow arrows). (b) Operative images of superior surface of calcaneus following total resection, employing the heel splitting approach. Note the extensive conglomerates of black grains scattered over that surface (white solid arrows) and the articular cartilage erosions of the anterior and middle facets of the subtalar joint (hollow white arrows). Complete destruction of the articular cartilage of the posterior facet of the subtalar joint with gross deformation is depicted (hollow black arrow). (c) Operative images of inferior surface of the calcaneus following total resection, employing the heel splitting approach. Note the presence of multiple bony cavities of various sizes (white arrows). Foci of black granular discharge are depicted (hollow arrows). (d) Brown fungal colonies with surrounding inflammatory cells and fibrosis (H and E, ×400).

In march 2015, we performed total calcanectomy through a heel splitting approach. Under general anesthesia, a high tourniquet was employed in the prone position. Anatomic pathology of the excised calcaneus is depicted in Figures 2(a), 2(b), 3(a), 3(b), and 3(c). The histopathological features were consistent with eumycetoma (Figure 3(d)). The patient was put on oral Itraconazole therapy for 8 weeks. Oral clindamycin therapy was used to treat secondary bacterial infection. Significant clinical improvement was observed at regular followup intervals. The discharging sinuses and the surgical wound demonstrated sound healing.

## 3. Discussion 

The concept of radiologic-pathologic correlation was born in 1947 with the establishment of the Radiologic Pathology Department and Registry at the Armed Forces Institute of Pathology in Washington, DC. This method has become a key teaching tool to understand the radiological manifestations of disease, initially on plain films and later with cross-sectional techniques [5]. Obviously, the most suitable candidate for radiologic-pathologic correlation is the study of neoplasms, since they are resected ideally in toto and therefore offer excellent imaging-gross pathologic comparisons.

Musculoskeletal infection is common in clinical practice. Osteomyelitis refers to infection of the bone and marrow usually by pus producing organisms. It is commonly caused by bacteria, but fungi are also considered. Usually, the diagnosis of fungal osteomyelitis is challenging. The clinical and radiological findings of osteoarticular infection may be conflicting and confusing. Even with the advancement of imaging technology, findings may remain inconclusive. There are a wide variety of presentations seen on imaging reflecting the balance between host and organism, disease duration, and institution of antimicrobial treatment. A good understanding of various stages of osteoarticular infection is essential to augment the interpretation of the vast amount of imaging information available to arrive at an accurate diagnosis and thereby institute timely and effective treatment to prevent destructive sequelae in the immature skeleton [5].

In eumycetoma fungal osteomyelitis bones are almost always attacked from outside, in contrast to bacterial osteomyelitis, and periosteal reaction, cortical erosions, and bone texture abnormalities may then be seen. It is important to detect whether bone is infected as nonsurgical cure is uncertain in such cases. Conventional radiographs are used to determine whether bone is affected and to identify the limits of lesions in eumycetoma. Multiple radiographic changes can be detected [3]. Abd El Bagi suggested a radiographic classification of mycetoma to determine the extent of lesions based on radiographic records of 516 patients seen in the Mycetoma Research Centre, Khartoum, Sudan [3]. Radiographs of our patient depicted soft tissue involvement, cortical erosion, and central cavitation of the calcaneus with minimal involvement of the talus and are classified as stage IV. These findings indicate the extent and severity of infection in our patient.

Several authors found that MRI is useful for visualizing soft tissue involvement and bone destruction; with MRI, multiple small spherical hyperintense lesions separated by tissue of low signal intensity appear. Some of these lesions show a central tiny hypointense focus. These hypointense foci were named “dot in circle” and were seen in 80% of patients, which made this appearance highly indicative of mycetoma [2, 4, 6, 7]. We were able to demonstrate similar MRI findings in all image sequences in our case. We were also able to clearly correlate the hypointense foci with the massive black fungal grains noticed on growth pathological examination of the calcaneus following total calcanectomy. In addition, we suggest that these characteristic (dot-in-circle) lesions may differentiate mycetoma from other infections and tumorous lesions.

The MRI of our patient revealed thickening of the overlying skin with multiple defects, ulceration, and sinus tracts breaching the posterior and plantar posterior skin, subcutaneous fat, and plantar fascia. These findings clearly correlated with the intraoperative multiple small cavities and black grain discharge observed on the plantar surface of the calcaneus following calcanectomy. There was a significant correlation between the previous radiologic-pathologic findings and the black grain discharging sinuses observed on clinical examination.

The MRI findings of our patient revealed markedly abnormal signal intensity involving the sinus tarsi and the subtalar articulation. The gross pathologic findings of the calcaneus revealed complete destruction of the articular cartilage of posterior subtalar joint, joint depression, and partial destruction of the middle facet. There was a significant correlation between the previous radiologic-pathologic findings and the preoperative painful passive range of motion of the subtalar joint.

In 2012, El Shamy and colleagues reported a new grading system for MRI diagnosis of mycetoma. They found that actinomycetoma more often showed soft tissue microabscesses, bony periosteal reaction, and reactive sclerosis, while eumycetoma frequently exhibited soft tissue macroabscesses with bone cavitation; such differences were not statistically significant [8]. The MRI findings of eumycetoma in our patient typically correlated with the eumycetoma findings presented by the previous authors. Additionally, there was a correlation between MRI findings in our patient and both the gross and histopathological findings that revealed bone cavitation and intervening abscesses full of brown fungal colonies (eumycetoma).

### 3.1. Anatomic-Histopathological Correlation

Since both actinomycetes and fungi are implicated as causative agents, it is important to distinguish them in order to ensure that correct treatments are given [9]. Mycetoma is characterized by the development of discharging sinuses. Within the discharged material, the causative organism is located both in and outside the grains. The colours of the grains can often be indicative of the aetiological agent: fungal grains are usually black or pale, while those of actinomycetes are white, yellow, or red [9].

Culture methods are still considered to be the gold standard in species identification of the causal agents of mycetoma. However, some agents are difficult to identify by morphology alone. Furthermore, most cultures methods are time consuming, contamination is common, and experience is needed to read results accurately [10]. Histology and cytology appeared to be not specific enough to identify the causative agent to the species level [10]. In the current study, black granular discharge was demonstrable on the surface of and inside cavities of the gross specimen of the calcaneus. These findings are consistent with eumycetoma.

The granules of actinomycetoma consist of fine, branching filaments, only about 1 micron thick, whereas the granules of eumycetoma are composed of septate hyphae 4-5 microns thick [11]. This histopathological description of eumycetoma conforms to that reported in our histograms. Furthermore, eumycetoma grains can be divided into black and pale grains. In histology sections, two types of grains of this agent are seen: filamentous and vesicular. The filamentous type is the most common and consists of brown septate and branched hyphae [10]. The histograms of our patient correlated to the detailed description of the filamentous type.

In our patient, all of the above mentioned histopathological findings clearly correlated to the anatomic pathology aiding definitive species diagnosis.

## 4. Conclusion

Agreement between radiology and pathology was recognizable. The radiologic-pathologic correlation may be used in explaining the role of plain radiographs and MRI in identifying eumycetoma osteomyelitis of the calcaneus in children. Establishment of a clear correlation between radiology and pathology may help differentiate eumycetoma from other infections and tumorous lesions. Radiologic-pathologic correlation augmented diagnostic confidence. Similarly, anatomic-histopathological correlations consolidated diagnostic accuracy.
